# Food Insecurity, Memory, and Dementia Among US Adults Aged 50 Years and Older

**DOI:** 10.1001/jamanetworkopen.2023.44186

**Published:** 2023-11-21

**Authors:** Haobing Qian, Aayush Khadka, Suzanna M. Martinez, Sonali Singh, Willa D. Brenowitz, Adina Zeki Al Hazzouri, Tanisha G. Hill-Jarrett, M. Maria Glymour, Anusha M. Vable

**Affiliations:** 1 Department of Family and Community Medicine, University of California, San Francisco; 2 Department of Epidemiology and Biostatistics, University of California, San Francisco; 3 Kaiser Permanente Center for Health Research, Portland, Oregon; 4 Department of Epidemiology, Mailman School of Public Health, Columbia University, New York, New York; 5 Global Brain Health Institute, University of California, San Francisco; 6 Department of Epidemiology, Boston University, Boston, Massachusetts

## Abstract

**Importance:**

Despite existing federal programs to increase access to food, food insecurity is common among US older adults. Food insecurity may affect Alzheimer disease and Alzheimer disease–related dementias via multiple mechanisms, yet there is almost no quantitative research evaluating this association.

**Objective:**

To examine whether food insecurity in older adults is associated with later-life cognitive outcomes.

**Design, Setting, and Participants:**

This cohort study of US residents aged 50 years and older from the US Health and Retirement Study was restricted to respondents with food insecurity data in 2013 and cognitive outcome data between calendar years 2014 and 2018. Analyses were conducted from June 1 to September 22, 2023.

**Exposure:**

Food insecurity status in 2013 was assessed using the validated US Department of Agriculture 6-item Household Food Security Module. Respondents were classified as being food secure, low food secure, and very low food secure.

**Main Outcomes and Measures:**

Outcomes were dementia probability and memory score (standardized to 1998 units), estimated biennially between 2014 and 2018 using a previously validated algorithm. Generalized estimation equations were fit for dementia risk and linear mixed-effects models for memory score, taking selective attrition into account through inverse probability of censoring weights.

**Results:**

The sample consisted of 7012 participants (18 356 person-waves); mean (SD) age was 67.7 (10.0) years, 4131 (58.9%) were women, 1136 (16.2%) were non-Hispanic Black, 4849 (69.2%) were non-Hispanic White, and mean (SD) duration of schooling was 13.0 (3.0) years. Compared with food-secure older adults, experiencing low food security was associated with higher odds of dementia (odds ratio, 1.38; 95% CI, 1.15-1.67) as was experiencing very low food security (odds ratio, 1.37; 95% CI, 1.11-1.59). Low and very low food security was also associated with lower memory levels and faster age-related memory decline.

**Conclusions and Relevance:**

In this cohort study of older US residents, food insecurity was associated with increased dementia risk, poorer memory function, and faster memory decline. Future studies are needed to examine whether addressing food insecurity may benefit brain health.

## Key Points

**Question:**

Is food insecurity among older adults associated with higher subsequent dementia risk and memory decline?

**Findings:**

In this cohort study of 7012 older adults, food insecurity was associated with an increased estimated dementia risk. Food insecurity was also associated with lower memory scores and faster memory decline.

**Meaning:**

The findings of this study suggest that food insecurity among older adults is associated with worse cognitive performance and higher dementia risk.

## Teaser

This cohort study examines cognitive outcomes in older individuals with food insecurity.

## Introduction

The number of US residents aged 65 years and older living with Alzheimer disease and Alzheimer disease–related dementias (AD/ADRD) is expected to increase from 5.8 million in 2020 to 14 million by 2060.^1^ Similarly, food insecurity, defined as a lack of consistent access to enough food for a healthy, active lifestyle, is persistent, and the prevalence in households with elderly individuals increased from 5.3% (2001) to 7.1% (2021).^2,3,4^ Among adults between the ages of 50 and 59 years, the prevalence of food insecurity is estimated to be higher at 9.4% in 2021.^5^ Older adults living with food insecurity are more likely to have lower nutrient intakes and experience poorer health outcomes, such as cardiovascular and metabolic diseases, increased stress and depression, and increased dementia risk.^6,7,8,9,10^

**Figure 1.**
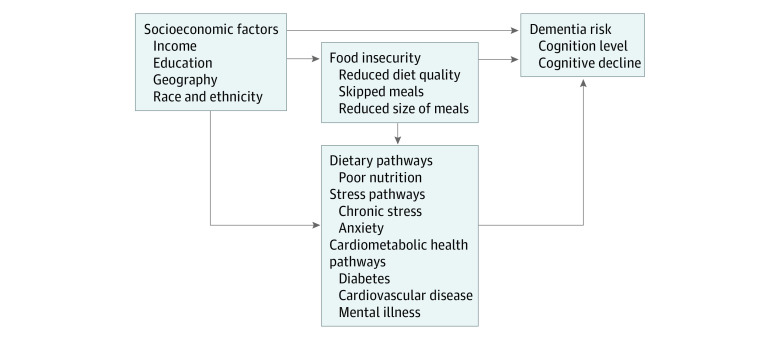
Hypothesized Mechanisms Linking Food Insecurity With Dementia and Cognitive Outcomes Multiple pathways through which food insecurity may plausibly impact later-life cognitive outcomes and risk of dementia.

The Lifecourse Health Development Framework, which explains how health trajectories develop over a lifetime, informs several plausible mechanisms by which food insecurity impacts dementia risk (Figure 1).^11^ First, food insecurity arises due to financial constraints, which limit access to healthful foods and contribute to a lower quality, quantity, and variety diet.^12,13^ Next, food insecurity may lead to poor nutrition, trigger stress pathways, or increase the likelihood of poor cardiometabolic health and mental illness. Ultimately, these factors, including food insecurity, may increase the risk of cognitive decline.^9,14,15,16,17,18^

Few studies have rigorously investigated food security in terms of its association with AD/ADRD. Earlier studies on this topic have been conducted primarily on cross-sectional data with small and selected subpopulations or have used inconsistent measures of food insecurity and later-life AD/ADRD risk.^19^ A recent systematic review^20^ identified only 1 longitudinal study examining food insecurity’s association with subsequent cognitive decline in older adults, with an association over a 2-year follow-up, consistent with several cross-sectional studies that also documented associations between food insecurity and cognitive decline.^21,22,23,24,25^

As food insecurity may be modifiable through existing government programs (eg, the Supplemental Nutrition Assistance Program [SNAP]) and there are limited existing treatment options for dementia, it is important to evaluate whether food insecurity is associated with increased dementia risk.^26,27^ We rigorously evaluated the association between food insecurity in later life and cognitive health, including dementia risk and age-related memory decline, in a large and diverse sample of US older adults. Our work builds on prior literature by using longitudinal data, validated measures of food insecurity and dementia risk, and robust adjustment for life course socioeconomic variables.

## Methods

### Sample

This cohort study used data from the Health and Retirement Study (HRS), a nationally representative, biennially fielded longitudinal survey of noninstitutionalized individuals aged 50 years and older and their spouses.^28^ The HRS and its various substudies collect rich data on life course demographic characteristics, health, labor market, and socioeconomic status. This study was waived from institutional board review because it did not involve human participation by the Human Research Protection Program at the University of California, San Francisco. This study followed the Strengthening the Reporting of Observational Studies in Epidemiology (STROBE) reporting guideline.

We used food security status data from the 2013 Health Care and Nutrition Study (HCNS), an HRS substudy regarding health care access, food purchases, food consumption, and nutrition (N = 8073).^29^ We combined HCNS data with outcome and covariate data from the broader HRS survey. Our study period was from calendar years 2013 to 2018.

Among HCNS participants, we excluded those younger than 50 years (n = 176, typically spouses of age-eligible participants) or those not included in the HRS cross-wave respondent tracker data set (n = 2). We also excluded participants who did not provide complete data on food security status (n = 501) or had missing cognition data for the entire study period (n = 382).

### Exposure

Food security status was assessed using the validated US Department of Agriculture (USDA) 6-Item Short Form US Household Food Security Survey Module.^30^ Respondents were asked a series of questions related to food purchases and consumption over the past 12 months to determine food security status, such as whether they were able to afford the food they needed or whether they ate less due to financial constraints (full questionnaire presented in eTable 1 in Supplement 1). Using USDA guidelines, we summed the number of affirmative answers to the 6 questions (score range, 0-6), where higher raw scores indicate higher food insecurity levels. We then categorized the total score into 3 levels in accordance with the USDA guidelines: food secure (high and marginal statuses, scores: 0-1), low food secure (scores: 2-4), and very low food secure (scores: 5-6).

### Outcomes

The outcomes were algorithmically defined and previously validated dementia probability and memory scores measured in the 2014, 2016, and 2018 waves of the HRS.^31^ Both measures were developed specifically in the HRS by training prediction models on the Aging, Demographics, and Memory Study (ADAMS) substudy. The ADAMS substudy focused on HRS respondents aged 70 years and older in 2000 and 2002 and had participants go through a full neuropsychological battery and dementia diagnosis.^32^

Dementia probability is the risk of a respondent being demented at the time of the survey. It is a composite measure that combines responses to various cognitive questions in the HRS as well as demographic information.^31,33^ Dementia probability values range from 0 to 1, and higher values reflect higher dementia risk. Compared with the standard clinical diagnosis of dementia, the dementia probability algorithm achieved a C statistic of 0.67, indicating acceptable performance.

Memory score is a measure of a respondent’s memory-related cognitive function at the time of the survey. It is also a composite measure that combines responses from various cognitive questions and demographic information. Higher values of memory score reflect better memory performance. We standardized memory score in our sample to the 1998 memory score distribution in the HRS for ease of interpretation.

### Covariates

We adjusted for the following potential confounders: age at food security assessment (in 2013), sex (male and female), self-reported race and ethnicity as part of the HRS surveys (non-Hispanic Black, Hispanic, non-Hispanic White, and Other), educational level (in years, linear and quadratic terms), birthplace (by census regions), married or not married status (in 2012, the year preceding the food security assessment), age at each interview wave (centered at 70 years, linear and quadratic terms), and body mass index (self-reported, 2012). Race was included as a proxy for racialized experiences that structurally minoritized groups face, which is associated with both the exposure and outcome. The Other race category included non-Hispanic categories of Alaska Native, American Indian, Asian, Native Hawaiian, Pacific Islander, and any other self-specified race.

We also included flexible specifications of several baseline (2012) measures of income and wealth to account for confounding by socioeconomic status: total earnings; total wealth; total assets, including second home; poverty status (yes or no); mother’s and father’s years of education (in years, linear and quadratic terms); labor force status (yes or no); home ownership (categorical variable: own or buying, renting, or living rent-free with relative, employer, friends, or others), veteran status (yes or no); Social Security income; welfare benefits; veteran benefits; and SNAP benefits the household received. As these socioeconomic status measures may all be correlated, we examined the covariance matrix to evaluate collinearity and found that no confounders were correlated above 0.8. All income, wealth, and social benefits were inflation adjusted to 2018 US dollars. When covariate data were missing in 2012, we used the 2010 value instead. We also created missing indicators (1 = data missing, 0 = otherwise) for mother’s educational level and father’s educational level as missing parental education data may indicate that the respondent did not reside with their parents during childhood, which is a marker of social capital.^34,35^ To minimize the possibility that those with lower cognition are less likely to apply for SNAP benefits, we additionally controlled for preexposure cognition, ie, dementia risk and memory score in 2012.^36^

### Statistical Analysis

To reduce selection bias due to missing data, we assumed the missingness mechanism was missing at random and used multiple imputation chained equation to impute missing covariates (558 [7.1%] individuals for dementia risk; 556 [7.0%] individuals for memory score) and outcome data (382 [5.4%] for the entire study period, 157 [2.2%] for baseline dementia risk, and 154 [2.2%] for baseline memory score).^37^ We constructed 10 imputed data sets. In each imputed data set, we estimated inverse probability weights to address censoring due to death (details in the eMethods in Supplement 1).^38,39^

In each imputed data set, we estimated the food insecurity and dementia risk association using generalized estimating equations specifying an independent correlation structure and applying robust SEs to account for repeated-outcome measures.^40,41,42,43,44,45^ Similarly, in each imputed data set, we estimated the food insecurity and memory association using a linear mixed-effects model. We modeled the food insecurity association with age-related memory decline, using an interaction between the exposure and age. We used Rubin rules to combine estimates across all imputed data sets.^46^

After combining the estimates, we contextualized the food insecurity association with memory by dividing coefficients on the exposure variable by the coefficient on the age covariate in our model to translate our primary associations into years of excess cognitive aging. We plotted predicted memory values by age and food insecurity status to visualize the food insecurity and memory association.

To test whether our results were robust to different exposure specifications, we redefined food insecurity status using alternative USDA cut points: first, as a binary indicator for food secure and insecure status (food secure = high or marginal food security; food insecure = low and very low food security), and second, as a 4-level categorical variable for food secure status (high vs marginal vs low vs very low).

We also tested whether our results were robust to different outcome specifications. For dementia probability, we used 3 additional algorithmically defined measures of dementia risk developed in the HRS data by Gianattasio et al.^47^ These were based on a modified Hurd algorithm, an algorithm developed using expert input to select covariates, and a machine learning–based least absolute shrinkage and selection operator–reduced logistic regression algorithm. Data on all 3 dementia risk measures were available for direct download from the HRS website. For memory score, we used immediate and delayed word recall as alternative outcomes. These scores were not algorithmically defined and were only available for direct respondents to the HRS.

Additionally, we refit our primary models by excluding SNAP benefits as a covariate. This is because SNAP benefits could potentially be endogenous with the food insecurity exposure, as there may be some temporal overlap between SNAP receipt status and the 1-year period during which food insecurity is measured. We also conducted the primary analyses in the complete case sample to assess our method of handling missing data. To acknowledge that adjusting for preexposure cognition may introduce regression-to-the-mean bias in change score analysis, we also performed sensitivity analyses without adjusting for the 2012 memory score.^48^

Regression models were adjusted for age at baseline, age centered at 70 years (linear and quadratic terms), 2012 dementia risk, sex, race and ethnicity, years of education, mother’s education, father’s education (linear and quadratic terms), birthplace, marital status, self-reported body mass index, income and wealth (linear and quadratic terms), poverty status, labor force status, home ownership, amount received from food stamps, welfare benefits, veteran status, veteran benefits, and Social Security income. Regression model parameters were estimated after imputing the primary analytic sample 10 times to fill in missing values using inverse probability of censoring weights to account for potential differential attrition and combining the results from all imputed data sets using the Rubin rules.

All analyses were conducted in Stata MP, version 18 (StataCorp LLC). One of us (A.K.) reviewed the entire code as is recommended practice.^49^ Analyses were conducted from June 1 to September 22, 2023. We report 2-sided 95% CIs. All statistical tests were 2-sided, with a significance threshold of *P* < .05.

## Results

Our primary analytic sample included 7012 participants (18 356 person-waves; mean person-waves per respondent, 2.6). Respondents’ mean (SD) age was 67.7 (10.0) years, 4131 (58.9%) were women, 2881 (41.1%) were men, 1136 (16.2%) were non-Hispanic Black, 4849 (69.2%) were non-Hispanic White, 4357 (62.4%) were married in 2012, and mean (SD) duration of schooling was 13.0 (3.0) years (Table). Overall, 18.4% of the analytic sample were food insecure: 10.3% experienced low food security and 8.1% experienced very low food security. In the analytic sample, approximately 11% of individuals aged 65 years or older at baseline reported being food insecure. In contrast, approximately 28% of individuals younger than 65 years in our analytic sample reported being food insecure. Compared with those who were food secure (marginal or high), those with low and very low food security were younger, more likely to be women and non-Hispanic Black or Hispanic, had fewer years of schooling, lived in poverty, earned less, received greater welfare support, and were renters. Respondents experiencing low and very low food security were also less likely to be married.

**Table.** Baseline Characteristics by Food Security Status in the Primary Analytic Sample^a^

Characteristic	Overall (N = 7012)	Food security			*P* value
		High and marginal (n = 5719 [81.6%])	Low (n = 725 [10.3%])	Very low (n = 568 [8.1%])	
**Outcome measures**					
Dementia risk in 2014, mean (SD)	0.1 (0.2)	0.1 (0.2)	0.1 (0.2)	0.1 (0.2)	.06
Standardized memory score in 2014, mean (SD)	−0.1 (0.9)	−0.1 (0.9)	−0.1 (0.8)	0.1 (0.7)	<.001
**Covariates**					
Age in 2013, mean (SD), y	67.7 (10.0)	68.7 (10.0)	64.5 (9.4)	61.8 (7.6)	<.001
Sex, No. (%)					
Female	4131 (58.9)	3316 (58.0)	456 (62.9)	359 (63.2)	<.001
Male	2881 (41.1)	2403 (42.0)	269 (37.1)	209 (36.8)	<.001
Years of education, mean (SD)	13.0 (3.0)	13.4 (2.7)	11.1 (3.6)	11.7 (3.3)	<.001
Race and ethnicity, No. (%)^b^					
Hispanic	795 (11.3)	471 (8.2)	185 (25.5)	139 (24.5)	<.001
Non-Hispanic Black	1136 (16.2)	759 (13.3)	215 (29.7)	162 (28.5)	.22
Non-Hispanic White	4849 (69.2)	4325 (75.6)	290 (40.0)	234 (41.2)	<.001
Other	232 (3.3)	164 (2.9)	35 (4.8)	33 (5.8)	.59
Southern birth, No. (%)	2314 (57.0)	1795 (51.0)	283 (88.4)	236 (78.2)	<.001
Married in 2012, No. (%)	4357 (62.4)	3764 (66.1)	360 (50.0)	233 (41.1)	<.001
Years of schooling, mean (SD)					
Maternal	10.2 (3.6)	10.6 (3.3)	8.5 (4.1)	9.2 (4.1)	<.001
Paternal	10.0 (3.7)	10.3 (3.5)	8.6 (4.0)	9.0 (4.0)	<.001
In labor force in 2012 (yes vs no), No. (%)	3018 (43.3)	2451 (43.1)	320 (44.4)	247 (43.5)	<.001
Poverty status in 2012 (yes vs no), No. (%)	793 (11.4)	389 (6.8)	212 (29.4)	192 (33.8)	<.001
Veteran status in 2012 (yes vs no), No. (%)	793 (17.3)	389 (18.8)	212 (11.2)	192 (10.6)	<.001
Total wealth in 2012, mean (SD)	75 240.5 (113 795.5)	85 353.0 (122 996.2)	31 827.3 (31 452.8)	30 680.2 (35 757.1)	<.001
Earnings in 2012, mean (SD)	18 787.0 (42 603.3)	20 729.2 (45 797.5)	10 527.8 (19 475.4)	10 127.4 (25 651.4)	<.001
Total assets in 2012, mean (SD)	484 090.9 (1 136 305.7)	580 492.9 (1 235 835.9)	75 673.9 (272521.0)	52 346.4 (139 314.0)	<.001
Received food stamps since last interview wave (yes vs no), No. (%)	673 (9.6)	303 (5.3)	177 (24.5)	193 (34.0)	<.001
Amount of food stamps received among recipients, mean (SD)	123.8 (573.8)	61.8 (389.6)	324.1 (892.6)	480.9 (1128.0)	<.001
Amount of Social Security income received among recipients, mean (SD)	180.0 (1283.9)	87.5 (960.8)	517.2 (2081.3)	664.1 (2221.6)	<.001
Amount of veteran benefits received among recipients, mean (SD)	753.7 (5082.4)	846.1 (5477.3)	357.4 (2739.9)	345.6 (2750.4)	<.001
Amount of welfare benefits received among recipients, mean (SD)	19.0 (357.1)	3.9 (116.3)	66.2 (692.9)	108.0 (890.1)	.003
Self-reported BMI, mean (SD)	28.8 (6.0)	28.5 (5.8)	30.3 (6.3)	30.5 (7.3)	<.001
Residency status, No. (%)					
Own home	5284 (75.4)	4635 (81.0)	401 (55.3)	248 (43.7)	<.001
Rent home	1183 (16.9)	682 (11.9)	247 (34.1)	254 (44.7)	<.001
Live rent free with others	202 (2.9)	142 (2.5)	34 (4.7)	26 (4.6)	.90
Other	21 (0.3)	17 (0.3)	3 (0.4)	1 (0.2)	.18

Abbreviation: BMI, body mass index (calculated as weight in kilograms divided by height in meters squared).

^a^ The distribution presented is from the analytical sample before imputations.

^b^ All race and ethnicity designations were self-reported by Health and Retirement Study participants. Other races include the following non-Hispanic categories: Alaska Native, American Indian, Asian, Native Hawaiian, Pacific Islander, and any other self-specified race.

### Dementia Risk Results

**Figure 2.**
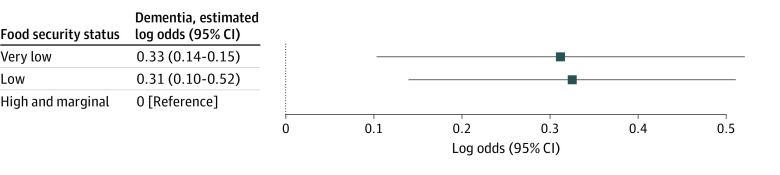
Low and Very Low Food Security Predict Elevated Dementia Risk in Primary Analytic Sample The analytical sample size is 7012 individuals, and 18 356 is the person-wave observations.

Compared with older adults with food security, those who experienced low food security had 1.38 times higher odds of dementia (95% CI, 1.15-1.67; in log odds: 0.33; 95% CI, 0.14-0.51), and those who experienced very low food security had 1.37 times higher odds of dementia (95% CI, 1.11-1.69; in log odds: 0.31; 95% CI, 0.10-0.52) (Figure 2; eTable 2 in Supplement 1). Translated to years of excess cognitive aging, point estimates showed that food insecurity is associated with increased dementia risk equivalent to approximately 1.3 excess years of aging.

### Memory Score Results

**Figure 3.**
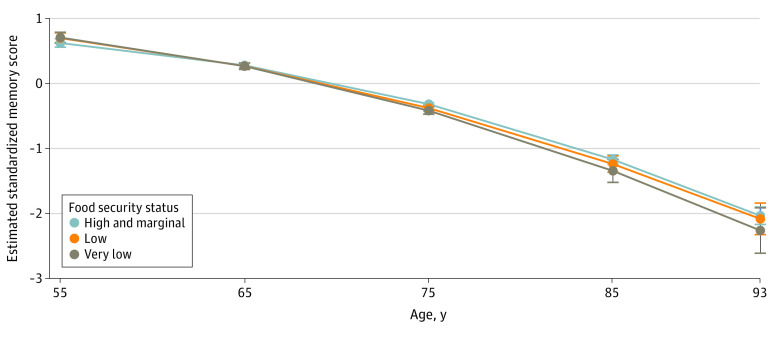
Estimated Memory Trajectories by Food Security Status in Primary Analytic Sample Estimates were made based on the regression model fit in the primary analysis. The analytical sample size is 7012 individuals, and 18 356 is the person-wave observations.

Compared with older adults with food security, those who experienced low and very low food security had worse memory levels at age 70 years (low β = −0.04; 95% CI, −0.08 to 0.00; very low β* = *−0.06; 95% CI, −0.1 to −0.01) (eTable 3 in Supplement 1). Translated to years of excess cognitive aging, low food insecurity was associated with decreased cognitive levels equivalent to approximately 0.7 years of excess aging per year, and very low food insecurity was associated with 1 year of excess aging per year. Older adults with food insecurity also had a marginally faster rate of age-related memory decline (low × age β = −0.005; 95% CI, −0.008 to −0.001; very low × age β = −0.009; 95% CI, −0.014 to −0.003) compared with food-secure individuals (eTable 3 in Supplement 1; Figure 3).

### Robustness Checks

Results were substantially similar (ie, point estimates and 95% CIs may vary, but conclusions are the same) under different exposure specifications (eTable 4 and eTable 5 in Supplement 1) and using different dementia algorithms (eTable 6 in Supplement 1) as well as immediate and delayed word recall scores (eTable 7 in Supplement 1). Estimates in models without adjusting for SNAP receipt (eTable 8 in Supplement 1), using complete case data (eTable 9 in Supplement 1), or without controlling for preexposure cognitive outcomes (eTable 10 in Supplement 1) were also quantitatively similar to the primary results.

## Discussion

This cohort study evaluated the association between food insecurity among older adults and subsequent cognitive health using validated measures from the large, diverse longitudinal US HRS. We found that individuals who experienced low or very low food security had a higher probability of dementia, worse memory level, and faster rate of memory decline compared with those who were food secure. Memory decline diverged slightly faster among the very low food security group than the low food security group. Our primary results were robust to alternative specifications of the exposure, outcome, and analytic models.

In the current study, 11% of HRS participants aged 65 years and older experienced food insecurity, which is approximately 4% higher than reported among older adult participants (age ≥60 years) in the 2020 Current Population Survey.^50^ The higher prevalence of food insecurity in our data was possibly a consequence of the Great Recession (2007-2009) in this population, which is consistent with examined trends in food insecurity.^51,52^ Current population survey data showed that nearly 9% of older adults experienced food insecurity with the onset of the Great Recession, with lingering effects into 2014.^53^ We also found that individuals experiencing food insecurity were, on average, younger and had lower educational attainment compared with those with food security. These findings suggest that food insecurity is more common among the same socioeconomically disadvantaged groups who are at high risk of dementia.^54,55,56,57^ Together, these findings highlight the role that social determinants of health may play later in life.

While much of the literature regarding food insecurity focuses on early life factors, our study is among the first highlighting the outcomes associated with food insecurity later in life.^24,58,59,60^ Recent findings by Lu and colleagues^61^ examined SNAP participation among SNAP-eligible adults—a group that is vulnerable to food insecurity—and found faster memory function decline among those experiencing food insecurity or not participating in SNAP compared with their counterparts. Our study examined food insecurity as the exposure, which builds on findings by Lu and colleagues given that SNAP participation among eligible older adults is underused compared with other segments of the population (48% compared with 86% overall in 2018).^36,62^

### Strengths and Limitations

Our study has strengths. Using a large and diverse data set, our study is in line with and adds evidence to the limited previous literature on food security and brain health by using both validated exposure and outcome data: the 6-item USDA Household Food Security Module and algorithmically defined, validated dementia probability and memory scores. Additionally, our outcome measures allowed us to incorporate information from both direct and proxy respondents in the HRS. Another strength is that food security was assessed before the outcome; therefore, we were able to examine exposure that temporally preceded the outcome.

Our study has limitations. A previous study using HRS data found that among SNAP-eligible adults, participants with reduced levels of cognitive function were less likely to participate in SNAP.^36^ However, in an advance over earlier work, we adjusted for preexposure cognition, minimizing this potential bias. Second, residual confounding remains a possibility in our study. To mitigate residual confounding, we included variables that reflected childhood and adulthood socioeconomic status and included flexible specifications of these variables. Weight loss in subclinical AD could exacerbate the effects of food insecurity on cognitive outcomes, although we adjusted for preexposure body mass index in 2012. Another potential confounder is food insecurity status before 2013, which was not available using the 6-item questionnaire. Future studies could examine food insecurity over a longer period. In addition, clinical diagnosis of dementia was not directly observed.

Our cohort study has several policy implications. The findings suggest that food insecurity remains high among older adults. Additionally, given limited treatment options for dementia, reducing its risk by targeting modifiable risk factors, such as food security, may be necessary. Food security may be easier to modify than other potentially modifiable risk factors, such as education and exercise, as it may be addressable through existing federal programs, such as SNAP.^63^ Increasing SNAP participation rates among older adults might constitute a viable population-level solution to reduce AD/ADRD disparities and improve brain health. The take-up rates of SNAP among eligible individuals varies substantially by state, ranging from 55% in Wyoming to an estimated 100% in a number of states, such as Oregon, Washington, and Illinois.^62,64^ In general, one reason for lower uptake is high administrative burden in completing paperwork among eligible participants, which suggests that simplifying the application process might increase the take-up rate, especially for those with any cognitive impairment.^65^

## Conclusions

In this cohort study, we found that food insecurity in older adulthood was associated with increased dementia risk and faster memory decline. Our study contributes to a limited literature by capitalizing on a large and diverse sample, validated exposure and outcome measures, and longitudinal data to robustly evaluate these associations, providing evidence in support of the connection between food insecurity in older adulthood and subsequent brain health. Our findings highlight the need to improve food security in older adults and that doing so may protect individuals from cognitive decline and dementia. Bolstering SNAP by making it easier for older adults who are SNAP eligible to apply could potentially mitigate the negative association food insecurity has with brain health.

## Notes

Supplement 1. **eTable 1.** Six-Item USDA Food Security Module, Assessed in HRS Respondents in 2013 **eTable 2.** Association of Food Insecurity With Dementia Risk in Primary Analytic Sample **eTable 3.** Association of Food Insecurity With Memory Levels and Age-Related Decline in Primary Analytic Sample **eTable 4.** Association of Food Insecurity With Dementia Risk Under Different Exposure Definitions **eTable 5.** Association of Food Insecurity With Memory Score Among Respondents Under Different Exposure Definitions **eTable 6.** Association of Food Insecurity With Dementia Risk Under Different Definitions of the Dementia Risk Outcome **eTable 7.** Association of Food Insecurity With Word Recall in Primary Analytic Sample **eTable 8.** Association of Food Insecurity With Cognitive Outcomes in Primary Analytic Sample Excluding SNAP Receipt From Control Variables **eTable 9.** Association of Food Insecurity With Dementia Risk and Memory Score in Complete Case Sample **eTable 10.** Association of Food Insecurity With Dementia Risk and Memory Score in Models Not Controlling for Cognitive Outcomes in 2012 **eMethods.** Construction of Censoring Weights

Supplement 2.
Data Sharing Statement
