# Supplementary material for: Food Insecurity, Memory, and Dementia Among US Adults Aged 50 Years and Older
Source: JAMA Netw Open. 2023 Nov 21;6(11):e2344186. doi: 10.1001/jamanetworkopen.2023.44186 (PMC10663972; doi:10.1001/jamanetworkopen.2023.44186)
Supplement: Supplement 1. — eTable 1. Six-Item USDA Food Security Module, Assessed in HRS Respondents in 2013 eTable 2. Association of Food Insecurity With Dementia Risk in Primary Analytic Sample eTable 3. Association of Food Insecurity With Memory Levels and Age-Related Decline in Primary Analytic Sample eTable 4. Association of Food Insecurity With Dementia Risk Under Different Exposure Definitions eTable 5. Association of Food Insecurity With Memory Score Among Respondents Under Different Exposure Definitions eTable 6. Association of Food Insecurity With Dementia Risk Under Different Definitions of the Dementia Risk Outcome eTable 7. Association of Food Insecurity With Word Recall in Primary Analytic Sample eTable 8. Association of Food Insecurity With Cognitive Outcomes in Primary Analytic Sample Excluding SNAP Receipt From Control Variables eTable 9. Association of Food Insecurity With Dementia Risk and Memory Score in Complete Case Sample eTable 10. Association of Food Insecurity With Dementia Risk and Memory Score in Models Not Controlling for Cognitive Outcomes in 2012 eMethods. Construction of Censoring Weights [file jamanetwopen-e2344186-s001.pdf]

Supplementary Online Content

Qian H, Khadka A, Martinez SM, et al. Food insecurity, memory, and dementia among US adults aged 50 years and older. *JAMA Netw Open*. 2023;6(11):e2344186. doi:10.1001/jamanetworkopen.2023.44186

eTable 1. Six-Item USDA Food Security Module, Assessed in HRS Respondents in 2013

eTable 2. Association of Food Insecurity With Dementia Risk in Primary Analytic Sample

eTable 3. Association of Food Insecurity With Memory Levels and Age-Related Decline in Primary Analytic Sample

eTable 4. Association of Food Insecurity With Dementia Risk Under Different Exposure Definitions

eTable 5. Association of Food Insecurity With Memory Score Among Respondents Under Different Exposure Definitions

eTable 6. Association of Food Insecurity With Dementia Risk Under Different Definitions of the Dementia Risk Outcome

eTable 7. Association of Food Insecurity With Word Recall in Primary Analytic Sample

eTable 8. Association of Food Insecurity With Cognitive Outcomes in Primary Analytic Sample Excluding SNAP Receipt From Control Variables

eTable 9. Association of Food Insecurity With Dementia Risk and Memory Score in Complete Case Sample

eTable 10. Association of Food Insecurity With Dementia Risk and Memory Score in Models Not Controlling for Cognitive Outcomes in 2012

eMethods. Construction of Censoring Weights

This supplementary material has been provided by the authors to give readers additional information about their work.

© 2023 Qian H et al. *JAMA Network Open.*

eTable 1 Six-Item USDA Food Security Module, Assessed in HRS Respondents In 2013

Questions	Response options
1. The food that (I/we) bought just didn't last, and (I/we) didn't have money to get more.	Often true<br>Sometimes true<br>Never true<br>DK or refused
2. (I/we) couldn't afford to eat balanced meals.	Often true<br>Sometimes true<br>Never true<br>DK or refused
3. In the last 12 months, since the last (name of current month), did (you/you or other adults in your household) ever cut the size of your meals or skip meals because there wasn't enough money for food?	Yes<br>No<br>DK
4. [IF YES ABOVE, ASK] [4] How often did this happen?	Almost every month<br>Some months but not every month<br>Only 1 or 2 months<br>DK
5. In the last 12 months, did you ever eat less than you felt you should because there wasn't enough money for food?	Yes<br>No<br>DK
6. In the last 12 months, were you ever hungry but didn't eat because there wasn't enough money for food?	Yes<br>No<br>DK

© 2023 Qian H et al. *JAMA Network Open.*

eTable 2 Association of food insecurity with dementia risk in primary analytic sample

	Odds Ratio	95% confidence interval
Ref: High or marginal food security		
Low food security	1.38	[1.15,1.67]
Very low food security	1.37	[1.11,1.69]
N (person waves)	7,012 (18,356 person waves)	

Note: All models are adjusted for age at baseline, dementia risk in 2012, age centered at 70 (linear and quadratic terms), gender, race/ethnicity, years of education, mother's education, father's education (linear and quadratic terms), birthplace, marital status, self-reported BMI, income and wealth (linear and quadratic terms), poverty status, labor force status, home ownership, amount received from food stamp, welfare benefits, veteran status, veteran benefits, and SSI income. We use multiple imputation chained equations to fill in missing values in covariates including censoring weights to account for attrition due to death and come from 10 multiple imputed datasets. The analytical sample size is 7012 individuals and is 18356 person-wave observations.

© 2023 Qian H et al. *JAMA Network Open.*

eTable 3 Association of food insecurity with memory levels and age-related decline in primary analytic sample

	Standardized unit change	95% confidence interval
Ref: High or marginal food security		
Association of low food security with memory levels	-0.04	[-0.08, 0.00]
Association of very low food security with memory levels	-0.06	[-0.10, -0.01]
Association of age with memory	-0.06	[-0.06, -0.06]
Association of age squared with memory	-0.0013	[-0.0014, -0.0011]
Interaction between low food security and age	-0.005	[-0.008, -0.001]
Interaction between very low food security and age	-0.009	[-0.014, -0.003]
Interaction between low food security and age squared	0.0002	[-0.0002, 0.0005]
Interaction between very low food security and age squared	0.0001	[-0.0004, 0.0005]
N (person waves)	7,012 (18,356 person waves)	

Note: All models are adjusted for age at baseline, memory score in 2012, age centered at 70 (linear and quadratic terms), gender, race/ethnicity, years of education, mother's education, father's education (linear and quadratic terms), birthplace, marital status, self-reported BMI, income and wealth (linear and quadratic terms), poverty status, labor force status, home ownership, amount received from food stamp, welfare benefits, veteran status, veteran benefits, and SSI income. We use multiple imputation chained equations to

© 2023 Qian H et al. *JAMA Network Open.*

fill in missing values in covariates including censoring weights to account for attrition due to death, and come from 10 multiply imputed datasets. The analytical sample size is 7012 individuals and is 18356 person-wave observations.

© 2023 Qian H et al. *JAMA Network Open.*

eTable 4 Association of food insecurity with dementia risk under different exposure definitions

Outcome	Specification 1: Food insecurity exposure defined as a 3-level categorical variable (primary specification)		Specification 2: Food insecurity exposure defined as an indicator variable	Specification 3: (4-level specification)		
Reference	High and marginal food security		Food secure	High food security		
Categories	Low food security	Very low food security	Food insecure	Marginal food security	Low food security	Very low food security
Odds ratio	1.38	1.37	1.38	1.29	1.45	1.45
95% confidence interval	[1.15, 1.67]	[1.11, 1.69]	[1.17, 1.62]	[1.04, 1.62]	[1.20, 1.76]	[1.17, 1.79]
N (person waves)	7,012 (18,356 person waves)					

Notes: Specification 1: food insecurity measured as a 3-level categorical variable (high and marginal food security, low food security and very low food security); specification 2: food insecurity measured as a binary indicator; specification 3: food insecurity measured 4-level categorical variable (high food security, marginal food security, low food security and very low food security). The exposure measure is a one-time measure among a subset of respondents who participated in the 2013 Health Care and Nutrition Study. All models are adjusted for age at baseline, dementia in 2012, age centered at 70 (linear and quadratic terms), gender, race/ethnicity, years of education, mother's education, father's education (linear and quadratic terms), birthplace, marital status, self-reported BMI, income and wealth (linear and quadratic terms), poverty status, labor force status, home ownership, amount received from food stamp, welfare benefits, veteran status, veteran benefits, and SSI income. We use multiple imputation chained equations to fill in missing values in covariates including censoring weights to account for attrition due to death and come from 10 multiply imputed datasets. The analytical sample size is 7012 individuals and is 18356 person-wave observations. The models are estimated using generalized estimating equations (GEE) with a logit link and independent correlation structure. Robust standard errors are applied. There is a dose-

© 2023 Qian H et al. *JAMA Network Open.*

response relationship in which higher food insecurity is associated with a higher level of elevated dementia risk (from specification 3). However, the reference group in specification 3 is a high food security group whereas the reference group includes high and marginal food security groups in the main specification and specification 2. Results should be interpreted with caution.

© 2023 Qian H et al. *JAMA Network Open.*

eTable 5 Association of food insecurity with memory score among respondents under different exposure definitions

**Specification 1 (High and marginal food security as the reference)**						
	Association of food insecurity with memory levels	95% confidence interval	Association of age with memory	95% confidence interval	Interaction between food insecurity and age	95% confidence interval
Low food security	-0.04	[-0.08, 0.00]	-0.06	[-0.06, -0.06]	-0.00	[-0.01, -0.00]
Very low food security	-0.06	[-0.10, -0.01]			-0.01	[-0.01, -0.00]
**Specification 2 (Food secure as the reference)**						
Food insecure	-0.05	[-0.08, -0.01]	-0.06	[-0.06, -0.06]	-0.01	[-0.01, -0.00]
**Specification 3 (High food security as the reference)**						
Marginal low food security	-0.05	[-0.10, 0.00]	-0.06	[-0.06, -0.05]	-0.01	[-0.01, -0.00]
Low food security	-0.05	[-0.09, -0.00]			-0.01	[-0.01, -0.00]
Very low food security	-0.06	[-0.11, -0.02].			-0.01	[-0.01, -0.00]
N (person waves)	7,012 (18,356 person waves)					

© 2023 Qian H et al. *JAMA Network Open.*

Notes: Specification 1: food insecurity measured as a 3-level categorical variable (high and marginal food security, low food security and very low food security); specification 2: food insecurity measured as a binary indicator; specification 3: food insecurity measured 4-level categorical variable (high food security, marginal food security, low food security and very low food security). The exposure measure is a one-time measure among a subset of respondents who participated in the 2013 Health Care and Nutrition Study. All models are adjusted for age at baseline, memory score in 2012, age centered at 70 (linear and quadratic terms), gender, race/ethnicity, years of education, mother's education, father's education (linear and quadratic terms), birthplace, marital status, self-reported BMI, income and wealth (linear term), poverty status, labor force status, home ownership, amount received from food stamp, welfare benefits, veteran status, veteran benefits, and SSI income. We use multiple imputation chained equations to fill in missing values in covariates including censoring weights to account for attrition due to death and come from 10 multiply imputed datasets. The analytical sample size is 7012 individuals and is 18356 person-wave observations. The models are estimated using linear mixed effects models. Robust standard errors are applied. The reference group in specification 3 is a high food security group whereas the reference group includes high and marginal food security groups in the main specification and specification 2. Results should be interpreted with caution.

© 2023 Qian H et al. *JAMA Network Open.*

eTable 6 Association of Food Insecurity with Dementia Risk under Different Definitions of the Dementia Risk Outcome

	Odds Ratio	95% confidence interval
**Outcome: Dementia risk defined using modified Hurd algorithm**		
Ref: High or marginal food security		
Low food security	1.30	[0.99, 1.70]
Very low food security	1.40	[1.00, 1.97]
N (person waves)	3,430 (6,022)	
**Outcome: Dementia risk defined using Expert model**		
Ref: High or marginal food security		
Low food security	1.25	[0.95, 1.64]
Very low food security	1.19	[0.86, 1.65]
N (person waves)	3,423 (5,965)	
**Outcome: Dementia risk defined using LASSO-reduced logistic regression**		
Ref: High or marginal food security		
Low food security	1.22	[0.97, 1.54]
Very low food security	1.07	[0.79, 1.46]
N (person waves)	3,348 (5,719)	

Notes: All models are adjusted for age at baseline, dementia risk in 2012, age centered at 70 (linear and quadratic terms), gender, race/ethnicity, years of education, mother's education, father's education (linear and quadratic terms), birthplace, marital status, self-reported BMI, income and wealth (linear and quadratic terms), poverty status, labor force status, home ownership, amount received

© 2023 Qian H et al. *JAMA Network Open.*

from food stamp, welfare benefits, veteran status, veteran benefits, and SSI income. We use multiple imputation chained equations to fill in missing values in covariates including censoring weights to account for attrition due to death, and come from 10 multiple imputed datasets.

© 2023 Qian H et al. *JAMA Network Open.*

eTable 7 Association of food insecurity with word recall in primary analytic sample

	Immediate Word Recall (ranging from 0-10)			Delayed Word Recall (ranging from 0-10)		
	Association of food insecurity with immediate word recall levels [95%CI]	Association of age with immediate word recall [95%CI]	Interaction between food insecurity and age [95%CI]	Association of food insecurity with delayed word recall levels [95%CI]	Association of age with delayed word recall [95%CI]	Interaction between food insecurity and age [95%CI]
Low food security	-0.10 [-0.23, 0.03]	-0.03 [-0.05, -0.02]	-0.00 [-0.01, 0.01]	-0.07 [-0.22, 0.08]	-0.04 [-0.05, -0.02]	0.00 [-0.01, 0.01]
Very low food security	-0.19 [-0.34, -0.05]		0.00 [-0.01, 0.01]	-0.17 [-0.35, 0.01]		-0.00 [-0.02, 0.01]
N (person waves)	7,012 (18,356 person waves)			7,012 (18,356 person waves)		

Notes: All models are adjusted for age at baseline, wore recall score in 2012, age centered at 70 (linear and quadratic terms), gender, race/ethnicity, years of education, mother's education, father's education (linear term), birthplace, marital status, self-reported BMI, income and wealth (linear and quadratic terms), poverty status, labor force status, home ownership, amount received from food stamp, welfare benefits, veteran status, veteran benefits, SSI income and total government transfer. We use multiple imputation chained equations to fill in missing values in covariates including censoring weights to account for attrition due to death and come from 10 multiply imputed datasets. The analytical sample size is 7012 individuals and is 18356 person-wave observations. The models are estimated using linear mixed effects models. The rate of decline for word recall may be biased towards the null due to non-response among the most cognitively impaired; this issue was avoided in our main results through using the measures developed by Wu and colleagues.

© 2023 Qian H et al. *JAMA Network Open.*

eTable 8 Association of Food Insecurity With Cognitive Outcomes in Primary Analytic Sample Excluding SNAP receipt from control variables

**Outcome: dementia risk**		
	Odds Ratio	95% confidence interval
Ref: High or marginal food security		
Low food security	1.38	[1.15, 1.67]
Very low food security	1.37	[1.11, 1.69]
**Outcome: memory score**		
	Standardized unit change	95% confidence interval
Ref: High or marginal food security		
Association of low food security with memory levels	-0.04	[-0.08, 0.00]
Association of very low food security with memory levels	-0.06	[-0.10, -0.01]
Association of age with memory	-0.06	[-0.06, -0.06]
Association of age squared with memory	-0.00	[-0.00, -0.00]
Interaction between low food security and age	-0.00	[-0.01, -0.00]
Interaction between very low food security and age	-0.01	[-0.01, -0.00]
Interaction between low food security and	0.00	[-0.00, 0.00]

© 2023 Qian H et al. *JAMA Network Open.*

age squared		
Interaction between very low food security and age squared	0.00	[-0.00, 0.00]
N (person waves)	7,012 (18,356 person waves)	

Notes: All models are adjusted for age at baseline, dementia and memory score in 2012, age centered at 70 (linear term), gender, race/ethnicity, years of education, mother's education, father's education (linear and quadratic terms), birthplace, marital status, self-reported BMI, income and wealth (linear and quadratic terms), poverty status, labor force status, home ownership, welfare benefits, veteran status, veteran benefits, and SSI income.

© 2023 Qian H et al. *JAMA Network Open.*

eTable 9 Association of food insecurity with dementia risk and memory score in complete case sample

**Outcome: dementia risk**		
	Odds Ratio	95% confidence interval
Ref: High or marginal food security		
Low food security	1.40	[1.15, 1.70]
Very low food security	1.48	[1.19, 1.84]
N (person waves)	6,454 (16,376 person waves)	
**Outcome: memory score**		
	Standardized unit change	95% confidence interval
Ref: High or marginal food security		
Association of low food security with memory levels	-0.05	[-0.10, -0.01]
Association of very low food security with memory levels	-0.06	[-0.10, -0.01]
Association of age with memory	-0.06	[-0.06, -0.06]
Association of age squared with memory	-0.00	[-0.00, -0.00]
Interaction between low food security and age	-0.00	[-0.01, -0.00]
Interaction between very low food security and age	-0.01	[-0.01, -0.00]

© 2023 Qian H et al. *JAMA Network Open.*

Interaction between low food security and age squared	0.00	[-0.00, 0.00]
Interaction between very low food security and age squared	0.00	[-0.00, 0.00]
N (person waves)	6,456 (16,382 person waves)	

Notes: All models are adjusted for age at baseline, dementia and memory score in 2012, age centered at 70 (linear term), gender, race/ethnicity, years of education, mother's education, father's education (linear and quadratic terms), birthplace, marital status, self-reported BMI, income and wealth (linear and quadratic terms), poverty status, labor force status, home ownership, amount received from food stamp, welfare benefits, veteran status, veteran benefits, and SSI income. The analytical sample includes 6,454 individuals (for dementia risk), 6,456 individuals (for memory score) and 16,376 person-wave observations (for dementia risk), 16,382 person-wave observations (for memory score) with complete information on covariates.

© 2023 Qian H et al. *JAMA Network Open.*

eTable 10 Association of food insecurity with dementia risk and memory score in models not controlling for cognitive outcomes in 2012

**Outcome: dementia risk**		
	Odds Ratio	95% confidence interval
Ref: High or marginal food security		
Low food security	1.40	[1.17, 1.68]
Very low food security	1.53	[1.26, 1.87]
**Outcome: memory score**		
	Standardized unit change	95% confidence interval
Ref: High or marginal food security		
Association of low food security with memory levels	-0.09	[-0.14, -0.03]
Association of very low food security with memory levels	-0.09	[-0.15, -0.03]
Association of age with memory	-0.06	[-0.06, -0.05]
Association of age squared with memory	-0.00	[-0.00, -0.00]
Interaction between low food security and age	-0.01	[-0.01, -0.00]
Interaction between very low food security and age	-0.01	[-0.02, -0.01]
Interaction between low food security and	0.00	[-0.00, 0.00]

© 2023 Qian H et al. *JAMA Network Open.*

age squared		
Interaction between very low food security and age squared	0.00	[-0.00, 0.00]
N (person waves)	7,012 (18,356)	

Notes: All models are adjusted for age at baseline, age centered at 70 (linear term), gender, race/ethnicity, years of education, mother's education, father's education (linear and quadratic terms), birthplace, marital status, self-reported BMI, income and wealth (linear and quadratic terms), poverty status, labor force status, home ownership, amount received from food stamp, welfare benefits, veteran status, veteran benefits, and SSI income.

© 2023 Qian H et al. *JAMA Network Open.*

eMethods: Construction of Censoring Weights

The weights are based on the inverse of the wave-specific probability of being observed at that wave (being alive and uncensored). The intuition is that respondents with similar characteristics to those missing due to attrition (censored) are upweighted.

For each wave of follow up, we define C_{it} as the censoring indicator whether individual i in wave t is no longer in the study (C_{it}=1). Each weight w_{it} is the reciprocal of the probability of remaining alive. We calculate the inverse probability weights for remaining uncensored using the following pooled logistic regression, using the same set of covariates as used in outcome analyses (noted previously):

$$logit\left(C_{it} \middle| fs_i, X_{1,i}, X_{2,i}\right) = \alpha_0 + \alpha_1 fs_i + \alpha_2' X_{1,i} + \alpha_3' X_{2,it} \qquad [1]$$

In Eq 1, fs_i refers to the food insecurity exposure variable, $X_{1,i}$ are time invariant covariates at the individual level, and $X_{2,it}$ are time varying covariates at the individual level. We used Eq 1 to predict the probability of remaining uncensored at time t $(1 - \Pr[C_{it} = 1])$ and then estimated the inverse of this probability:

$$w_{it}^{*} = \frac{1}{1 - \Pr\,[C_{it} = 1]} \qquad [2]$$

Finally, we estimated the individual level cumulative product of w_{it}^{*} to use as inverse probability of censoring weights in our regression models:

$$w_{it} = \prod_{t=1}^{T} w_{it}^{*} \qquad [3]$$

© 2023 Qian H et al. *JAMA Network Open.*
