# Supplementary material for: Food Insecurity, Memory, and Dementia Among US Adults Aged 50 Years and Older
Source: JAMA Netw Open. 2023 Nov 21;6(11):e2344186. doi: 10.1001/jamanetworkopen.2023.44186 (PMC10663972; doi:10.1001/jamanetworkopen.2023.44186)
Supplement: Supplement 2. — Data Sharing Statement [file jamanetwopen-e2344186-s002.pdf]

Data Sharing Statement

Qian. Food Insecurity, Memory, and Dementia Among US Adults Aged 50 Years and Older. *JAMA Netw Open*. Published November 21, 2023. doi:10.1001/jamanetworkopen.2023.44186

Data
Data available: Yes
Data types: Deidentified participant data
How to access data: The data used in this analysis are publicly available through the US Health and Retirement Study (HRS) website. Accessing these data requires opening a free account on the HRS website. The online link to the HRS data is: https://hrs.isr.umich.edu/data-products
When available: With publication

Supporting Documents
Document types: Statistical/analytic code
How to access documents: aayush.khadka@ucsf.edu
When available: With publication

Additional Information
Who can access the data: Data are publicly available through the US Health and Retirement Study website for anyone wishing to use them for their analyses.
Types of analyses: For research purpose.
Mechanisms of data availability: Data are publicly available through the US Health and Retirement Study website and can be accessed independently.
